# Mendelian randomization study revealed a gut microbiota-neuromuscular junction axis in myasthenia gravis

**DOI:** 10.1038/s41598-024-52469-7

**Published:** 2024-01-30

**Authors:** Jiaying Shi, Ming Yi, Shengyu Xie, Zhaokun Wang, Xinyue Zhang, Xiaolan Tan, Dachang Tao, Yunqiang Liu, Yuan Yang

**Affiliations:** Department of Medical Genetics, State Key Laboratory of Biotherapy, West China Hospital, Sichuan University, Chengdu, China

**Keywords:** Microbial genetics, Neurological disorders, Neuromuscular disease, Immunology, Microbiology, Neurology, Neurological disorders

## Abstract

A growing number of studies have implicated that gut microbiota abundance is associated with myasthenia gravis (MG). However, the causal relationship underlying the associations is still unclear. Here, we aim to investigate the causal effect of gut microbiota on MG using Mendelian randomization (MR) method. Publicly available Genome-wide association study (GWAS) summary-level data for gut microbiota and for MG were extracted. Inverse variance weighted was used as the main method to analyze causality. The robustness of the results was validated with sensitivity analyses. Our results indicated that genetically predicted increased phylum Lentisphaerae (OR = 1.319, *p* = 0.026), class Lentisphaerae (OR = 1.306, *p* = 0.044), order Victivallales (OR = 1.306, *p* = 0.044), order Mollicutes (OR = 1.424, *p* = 0.041), and genus Faecalibacterium (OR = 1.763, *p* = 0.002) were potentially associated with a higher risk of MG; while phylum Actinobacteria (OR = 0.602, *p* = 0.0124), class Gammaproteobacteria (OR = 0.587, *p* = 0.036), family Defluviitaleaceae (OR = 0.695, *p* = 0.047), family Peptococcaceae (OR = 0.698, *p* = 0.029), and family Family XIII (OR = 0.614, *p* = 0.017) were related to a lower risk of MG. The present study provides genetic evidence for the causal associations between gut microbiota and MG, thus suggesting novel insights into the gut microbiota-neuromuscular junction axis in the pathogenesis of MG.

## Introduction

Myasthenia gravis (MG) is a chronic autoimmune disease characterized by pathogenic antibodies directed against acetylcholine receptors (AChR), muscle-specific kinase (MUSK), lipoprotein-related protein 4 (LRP4), or other functionally related molecules in the postsynaptic membrane at the neuromuscular junction^1^. With a prevalence of 150 to 250 cases per million, MG forms the largest disease group of neuromuscular junction disorders, causing muscle weakness with fluctuations in severity and fatigability^2^. The diagnosis and subgroup stratification of MG are based on the comprehensive assessment of clinical symptoms and signs, positive test results for specific autoantibodies, neurophysiological examinations, identification of thymoma or thymic hyperplasia, or a characteristic response to therapy^1^. However, as the most important diagnostic method in the diagnosis of MG, antibody testing is relatively expensive, time-consuming, not always available, and has a high rate of false negatives^3^. Novel potential biomarkers with high specificity and sensitivity are needed to assist the diagnosis of MG for early interventions. In addition, few immunospecific therapies are available to date that are individualized for the MG patients to optimize effectiveness. Current symptomatic or nonspecific immunosuppression approaches for MG require long-term treatment and a drug combination with a high recurrence rate. Furthermore, an intensive care unit with intubation or noninvasive ventilation would be urgently needed when rapid worsening, severe weakness, or respiratory and cardiac failure occurs in 20% of MG patients, contributing to considerably high morbidity and mortality^4^. Therefore, another major challenge in MG management would be finding ambitious therapies that aim for full clinical and pharmacologic remission or minimal symptoms with near-normal physical function and high quality of life.

Gut microbiota, the specific microbial communities residing in the intestinal microecosystem, act as essential active components in a broad range of key physiological processes including nutrition synthesis, metabolism, and immune response regulation^5^. Numerous studies revealed that microbiota dysbiosis is strongly correlated with central (e.g. multiple sclerosis) and peripheral (e.g. systemic lupus erythematosus, type 1 diabetes, rheumatoid arthritis, Sjogren's syndrome, and celiac disease) autoimmune disorders^5–9^, providing insights into the critical roles of gut microbiota in the regulation of immune response. Recently, emerging evidence have suggested that patients with MG exhibit evident changes in the relative abundance of some gut microbial strains compared with healthy controls and indexes of α‐diversity were substantially different among different MG severity groups^10–15^, implying their potential roles as targets for novel diagnosis, severity prediction and therapeutics in MG. However, due to potential biases such as reverse causation or confounding factors in observational studies, the causal association between gut microbiota and MG has not been fully demonstrated. Randomized controlled studies are impractical and difficult to perform on this disease due to potential life-threatening risk and time-consuming follow-up^4^. Thus, elucidating whether the correlation is causal and identifying particular microbiome dysbiosis (characterized by altered diversity and composition) as well as the rise of pathobionts could guide clinical practice in MG management.

Mendelian randomization (MR) is a useful epidemiological approach that implies putative causal effects between exposures and outcomes using single nucleotide polymorphisms (SNPs) to construct instrumental variables (IVs)^16^. In contrast to conventional observational research, MR method exhibits evident advantages in eliminating confounding and reverse causation, because (1) alleles are randomly inherited to an individual at conception and the distribution of genetic variants that are associated with a particular risk factor is totally independent of environmental confounders; (2) reliable causal inferences could be obtained given that genetic variants are determined before particular phenotypes occur and cannot be modified by the progression of the disease outcome^16^. In this study, we conducted a two-sample MR analysis using publicly available genome-wide association study (GWAS) summary statistics to investigate the causal relationship between gut microbiota and MG. Our findings revealed a gut microbiota-neuromuscular junction axis in MG, which could provide reliable evidence for the gut microbiome representing potential fertile targets for MG diagnosis and drug development in clinical practice.

## Results

### Study design

**Figure 1:**
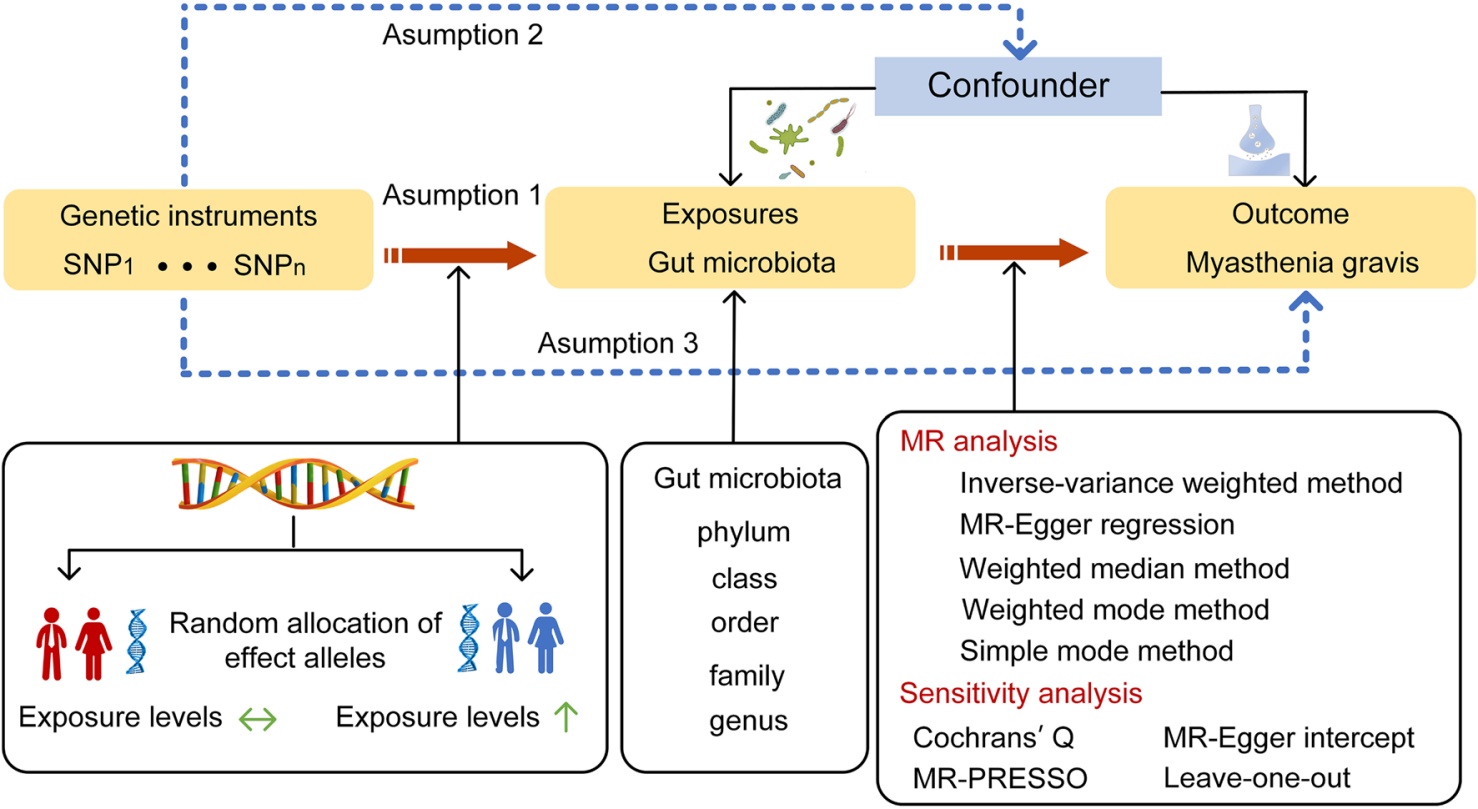
Study design overview. *SNP* single-nucleotide polymorphism, *MR* Mendelian randomization.

The causal association between 211 taxa and the risk of MG was systematically assessed by a two-sample MR design. The schematic representation of this study is presented in Fig. 1. Three fundamental assumptions should be met for causal estimates in a valid MR design: (1) IVs are strongly associated with exposures; (2) included IVs are not associated with any potential confounder; (3) IVs affect the outcome only through exposures of interest, ensuring the independence of horizontal pleiotropy^17^.

### MR estimations

**Figure 2:**
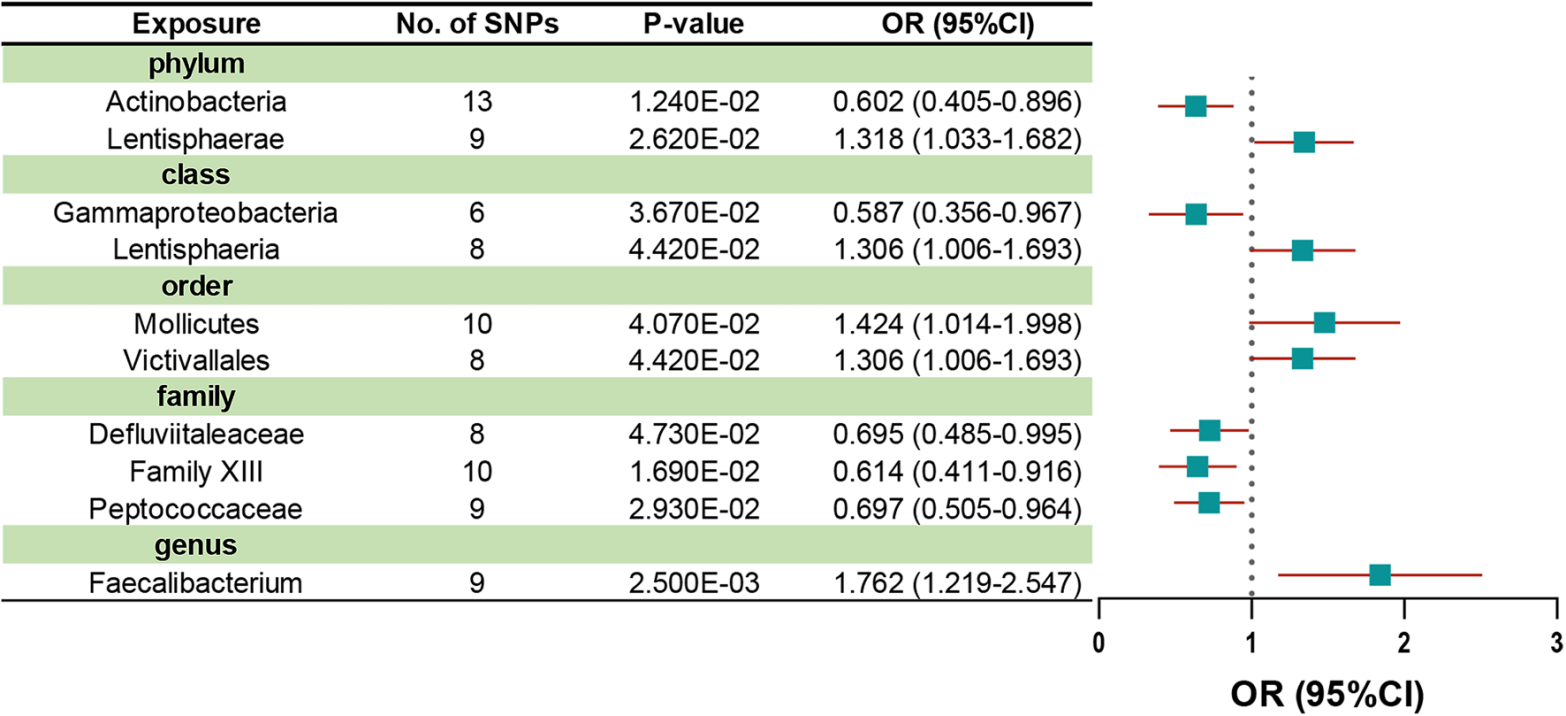
Significant and nominally significant MR estimates for the causal relationship between gut microbiota and MG using IVW method. *SNP* single nucleotide polymorphism, *OR* odds ratio, *95% CI* 95% confidence interval, *MR* Mendelian randomization.

After IV selection, 207 taxa (including 128 genera, 34 families, 20 orders, 16 classes and 9 phylum) were tested. The inverse variance weighted (IVW) method preliminarily identified 12 gut microbiota causally associated with MG (Fig. 2), two of which remained biologically unknown (unknown family id.1000005471 and unknown genus id.1000005472). Specifically, we found that host-genetic-driven increases in Lentisphaerae at phylum level (OR = 1.319; 95% CI = 1.033–1.683; *p* = 0.026), Lentisphaeria at class level (OR = 1.306; 95% CI = 1.007–1.694; *p* = 0.044); Victivallales at order level (OR = 1.306; 95% CI = 1.007–1.694; *p* = 0.044); Mollicutes at order level (OR = 1.424; 95% CI = 1.015–1.998; *p* = 0.041); and Faecalibacterium at genus level (OR = 1.763; 95% CI = 1.220–2.547; *p* = 0.002) were associated with higher risks of MG. Meanwhile, we found that genetically increases in Actinobacteria at phylum level (OR = 0.602; 95% CI  = 0.405–0.896; *p* = 0.012); Gammaproteobacteria at class level (OR = 0.587; 95% CI =  0.357–0.968; *p* = 0.036); Defluviitaleaceae at family level (OR = 0.695; 95% CI =  0.485–0.996; *p* = 0.047); Family XIII at family level (OR = 0.614, 95% CI =  0.412–0.916; *p* = 0.017); and Peptococcaceae at family level (OR = 0.698, 95% CI = 0.505–0.965; *p* = 0.029) were associated with lower risks of MG. After FDR correction (*P*_FDR_ < 0.1), phylum Lentisphaerae (*P*_FDR_ = 0.026), order Mollicutes (*P*_FDR_ = 0.089), order Victivallales (*P*_FDR_ = 0.089), and genus Faecalibacterium (*P*_FDR_ = 0.041) remained to be risk factors for MG, while phylum Actinobacteria (*P*_FDR_ = 0.012) remained to be a protective factor for MG. The MR results using MR-Egger, weighted median, simple mode, and weighted mode methods are shown in Supplementary Table 1, Supplementary Fig. S1.

The F-statistic for each IV for all analyses are shown in Supplementary Table 2. All F-statistics for the overall instruments were over 10, indicating a good strength of used genetic instruments.

### Sensitivity analyses

The results of sensitivity analyses are presented in Supplementary Table 3. No significant heterogeneity was observed in the Cochrane Q statistics (*p* > 0.05). Funnel plots showed that points representing causal effects of gut microbiota on MG were symmetrically distributed, indicating that causal associations were less likely to be affected by potential biases (Supplementary Fig. S2). The MR-Egger regression intercept approach revealed no evidence of horizontal pleiotropy (*p* > 0.05) (Supplementary Fig. S1). MR-PRESSO analysis indicated no outliers in the results. No single SNP strongly influenced the overall causal association effects in the leave-one-out sensitivity analysis (Supplementary Fig. S3).

Furthermore, we investigated the secondary traits of the gut microbiota-associated SNPs for further confounding analysis using Phenoscanner. The results showed that the selected SNPs associated with gut microbiota were not linked to any of the confounders.

## Discussion

There is a paucity of studies that identify the role of gut microbiota dysbiosis as a potential beneficial or harmful factor contributing to the occurrence and development of MG. In this study, we performed a large-scale MR analysis to comprehensively investigate the causal associations between gut microbiota and risk of MG. This study provides genetic evidence for the causal relationships of higher levels of phylum Lentisphaerae, class Lentisphaerae, order Victivallales, order Mollicutes, and genus Faecalibacterium with an elevated risk of MG; and higher levels of phylum Actinobacteria, class Gammaproteobacteria, family Defluviitaleaceae, family Peptococcaceae, and family Family XIII with a reduced risk of MG. Based on sensitivity analyses, no evidence of horizontal pleiotropy potentially influenced our results.

Victivallales is an order of bacteria in the class Lentisphaeria, phylum Lentisphaerae, Planctomycetes–Verrucomicrobia–Chlamydiae (PVC) superphylum. A decreased abundance of Lentisphaerae was observed in patients with post-traumatic stress disorder^18^, multiple sclerosis^19^, and autoimmune hepatitis^20^, whereas an increased abundance of Lentisphaerae was identified in Blastocystis-colonized children^21^ and rosacea patients^22^. Meanwhile, MR analyses demonstrated different effects of the host-genetic driven increase in Lentisphaerae on the risk of different diseases. For example, Ning et al. revealed suggestive causal effects of increased abundance of phylum Lentisphaerae, class Lentisphaeria, and order Victivallales on protective effects of Parkinson's disease^23^; while a greater abundance of Lentisphaerae was identified to be significantly associated with lower celiac disease risk^24^. In our study, an increased abundance of the three taxa was causally linked to an increased risk of MG, indicating their detrimental effects on MG. However, the causal results still need to be interpreted with caution and further investigations are needed to confirm our findings. Meanwhile, order Mollicutes was identified to be a risk factor for MG in our findings and a similar trend was also observed for Graves’ disease^25^. Given limited information in the literature, its specific function and links with MG require further investigations to reach any conclusive inferences.

Though phylum Actinobacteria represents only a small percentage in the four major phyla of the gut microbiota, accumulating evidence has suggested their beneficial effects in many pathological conditions. Actinobacteria was found to be significantly decreased in the fecal sample of MG patients compared with controls in several cross-sectional studies^12,26, 27^; while the inconsistent result showing increased abundance of Actinobacteria in the saliva of MG patients^28^ is expected to be attributed to the difference in distribution of intestinal and oral flora. Rinaldi et al. investigated the clinical efficacy of the therapeutic administration of vital bifidobacteria in MG and suggested that this probiotic therapy improved the symptoms of the experimental autoimmune MG model and decreased serum anti-rat AChR antibody levels^29^. Similarly, prophylactic or preventive bifidobacterial-based probiotic administration was also reported to ameliorate symptoms of mouse models and modulated AChR-specific immune responses^30,31^. Chen et al. demonstrated a significant restoration of the Actinobacteria abundance after administration of a traditional Chinese medicine in MG patients, who achieved remarkable symptom alleviation. The protective causal effect of Actinobacteria on MG identified in our study corroborated the findings of previous research and provided additional evidence for probiotic efficacy as supplementary therapy in MG.

Family Defluviitaleaceae, family Peptococcaceae, and family Family XIII belong to members of phylum Firmicutes which were found to be the main bacterial phyla reduced in MG patients^10,26^. The reduced ratio of Firmicutes/Bacteroidetes describes an inflammatory microbiota which could damage the intestinal epithelium, thereby triggering an immune response and leading to the immune imbalance in various autoimmune diseases^32,33^. Decreased abundance of Defluviitaleaceae and Peptococcaceae were reported in patients with autoimmune diseases such as systemic lupus erythematosus^34^, rheumatoid arthritis^35^, juvenile idiopathic arthritis^36^, and Graves’ disease^25^, while how they participate in the pathological process of MG remains unclear and requires further investigation. Surprisingly, our study identified genus Faecalibacterium, also a member of Firmicutes, as a risk factor for MG, though it was found to be depleted in patients with systemic lupus erythematosus, multiple sclerosis, and Sjögren’s syndrome in previous findings^37^. Evidence has suggested that Faecalibacterium could produce several metabolites such as pentanoate and butyrate in the intestine, leading to Treg cell activation and various anti-inflammatory activities with protective benefits^38,39^. Although MG is an autoimmune disorder characterized by reduced Treg cells, several studies showed similar results and indicated increased abundance of Faecalibacterium in MG patients than controls^14,26^. We speculate that the interplay of effects among different taxa in the refinement (e.g. level of genus and species) could have affected the observation of the impact. In addition, other variables could also be functionally connected in this causal association, either directly or indirectly by mediators in the complicated immune responses. Hence, this resulting correlation needs to be interpreted with caution, and more in-depth investigations are required to further elucidate the relationship.

Class Gammaproteobacteria belongs to phylum proteobacteria, the abundance and functional characterization of which have been mixed across studies. Devi et al. identified significant difference at the class level of the phylum Proteobacteria in the COVID 19 vaccination breakthroughs, where Alphaproteobacteria and Betaproteobacteria were found transcendent in the unvaccinated patients while Gammaproteobacteria was found enriched in the vaccinated patients^40^. Evidence exists to support this finding which suggests that an increase of Gammaproteobacteria produces beneficial effects for favorable immune responses post vaccination^41^. Moreover, several studies found a relatively lower abundance of Gammaproteobacteria in schizophrenia and demonstrated it was related to a lower risk for schizophreni^42–44^. In this study, we found that Gammaproteobacteria was associated with a decreased risk of MG, though different studies yielded conflicting results. Increased abundance of proteobacteria was observed in the fecal samples of MG patients compared with controls^10,14, 26^; whereas decreased abundance of Proteobacteria was reported in the saliva of MG patients^28^. Therefore, due to environmental confounders and heterogeneity in sample characteristics, research is needed to explore the role of specific class of proteobacteria phylum (Gammaproteobacteria in particularly) in MG.

Potential mechanisms of interactions between gut microbiota and MG have been investigated. Emerging evidence unraveled that the immunomodulatory effects of gut microbiota are mostly achieved through the Thl7/Treg axis^45^. Specifically, gut microbiota dysbiosis may lead to increased permeability of the intestinal mucosal barrier and an imbalance of Th17/Treg cells, consequently activating innate and downstream adaptive immune responses. Th17 cells could promote inflammatory responses by secreting IL-17, recruiting neutrophils, activating innate immune cells, and inducing release of a series of pro-inflammatory cytokines^46,47^. Meanwhile, Tregs could secret inhibitory cytokines and inhibit the function of other effector T cells and antigen-presenting cells, thereby suppressing immune responses^48^. In vivo experiments showed that IL-17 knock-out mice exhibited fewer or no MG symptoms and experienced remarkable reductions in pathogenic anti-murine AChR antibodies. These findings demonstrate that the secretion of IL-17 by CD4 + T cells contributes to the loss of B-cell tolerance and pathogenic antibody production, consequently leading to the classical antibody-mediated autoimmunity in MG^49^. Xu et al. observed significant reduction in the number and frequency of the activated Tregs (CD4 + CD25 + FOXP3 + Helios + T cells) in peripheral blood of the MG patients during active stage, concluding that decreased activated Tregs could be a critical contributor to the pathogenesis of MG^50^. Therefore, it’s tended to speculate that gut microbiota dysbiosis could contribute to the progression of MG by influencing the immune system activation and driving the pro- and anti-inflammatory responses through Thl7/Treg axis. Maintaining an appropriate Treg/Th17 balance, inducing the reconstruction of immune tolerance, and restoring homeostatic function might become a novel therapeutic strategy for MG management.

In conclusion, our findings indicated a gut-neuromuscular junction axis in MG development, thereby providing suggestive evidence for future drug development and guiding therapeutic agent selection to treat the disease. A similar investigation conducted by Su et al. also unveiled suggestive associations between several microbiota taxa and MG susceptibility^51^. However, summary statistics for outcome data in our study were derived from the currently largest meta-GWAS for MG (1873 MG patients and 36,370 controls), and Su et al.’s study data was obtained from a relatively small population from the FinnGen Research Project (426 MG cases and 373,848 controls). We anticipate that a larger population could provide greater generalizability of findings to the broader population, thereby increasing the external validity of the study. In addition, MG encompasses various subtypes, each characterized by distinct immunological and clinical features. These subtypes of MG underscore the heterogeneity of the disease and the diverse underlying immunological mechanisms. All 1873 patients included in our study exhibit AChR + MG, a subtype that constitutes the majority of MG cases. In contrast, the FinnGen Research Project contains various MG subtypes. Therefore, our study highlights a potential gut microbiota-neuromuscular junction axis in AChR + MG, necessitating tailored management strategies for this specific subtype.

Our study also had several limitations. Firstly, the majority of participants recruited in our study were of European ancestry, while a small number of data were taken from cohorts consisting of other races, which may partially bias our estimates. Secondly, the lowest taxonomic level in the exposure dataset was analyzed at the genus level but not at a more specialized level such as species or strain levels. Thirdly, GWAS summary statistics rather than raw data were used in the analysis, and subgroup analyses were not performed on such as early- and late-onset cases. Lastly, further prospective studies need to be conducted to clarify the underlying mechanism of such associations.

## Materials and methods

### Data sources

Summary statistics for exposure data were collected from the largest genome-wide meta-analysis of human gut microbiome published to date that was conducted by the international consortium MiBioGen (https://mibiogen.gcc.rug.nl/)^52^. Genotyping data of 18,340 individuals were collected from 24 cohorts which include samples from multiple ancestries (including European, Middle-Eastern, East Asian, American Hispanic/Latin, African American, etc.). Three distinct variable regions of the 16S rRNA gene were targeted to profile the microbial composition and a total of 211 taxa (131 genera, 35 families, 20 orders, 16 classes, and 9 phyla) were involved in this study. Summary statistics for outcome data were collected from the currently largest meta-GWAS for MG that was conducted in the US and Italy. This study enrolled 1873 MG patients with positive test results for AChR + antibodies and 36,370 neurologically normal individuals as controls^53^. Patients with positive test results for antibodies to anti-MuSK were excluded from enrollment.

### Instrumental variables (IVs) selection

A series of steps were performed as follows to select eligible IVs. Firstly, a less stringent cut-off of *P* < 1 × 10^−5^ was used to include more SNPs as IVs which were related to the human gut microbiome composition as reported^52,54^. Then, a linkage disequilibrium (LD) threshold of r^2^ < 0.001 over 10,000 kilobase pairs was used to select independent IVs in the clumping process based on the 1000 Genomes European reference panel^55^. SNPs with a minor allele frequency (MAF) ≤ 0.01 were removed. Furthermore, the resulting SNPs were extracted from the outcome data, and those significantly associated with outcome (*p* < 5 × 10^–5^) were excluded. The orientation of alleles was then inferred to align the exposure- and outcome-SNPs for harmonization. Palindromic SNPs (e.g. with A/T or G/C alleles) or SNPs with incompatible alleles (e.g. A/G vs. A/C) were discarded. In addition, all the identified SNPs were checked in PhenoScanner (www.phenoscanner.medschl.cam.ac.uk), a large curated database with comprehensive information regarding genotype–phenotype associations, to confirm the association between the SNPs and potential risk factors that might bias the MR estimates. Finally, gut microbiota with at least three shared SNPs were kept for MR analysis.

F-statistic was computed for each identified SNP to assess the statistical strength using the following formula as previously described^56^.

$$F = {{R^{2} \left( {N - 2} \right)} \mathord{\left/ {\vphantom {{R^{2} \left( {N - 2} \right)} {\left( {1 - R^{2} } \right)}}} \right. \kern-0pt} {\left( {1 - R^{2} } \right)}},$$

where R^2^ represents the proportion of the variability of the gut microbiome explained by each instrument; and N represents the sample size.

As previously described^56^, the following formula was used to calculate R^2^ for the 5 genome-wide significant SNP instrument,

$$R^{2} = 2 \times EAF \times \left( {1 - EAF} \right) \times beta^{2} ,$$

while another formula was used for the extended 10 SNP instrument,

$$R^{2} = \frac{{2 \times EAF \times \left( {1 - EAF} \right) \times beta^{2} }}{{\left( {2 \times EAF \times \left( {1 - EAF} \right) \times beta^{2} } \right) + \left( {2 \times EAF \times \left( {1 - EAF} \right) \times N \times SE\left( {beta} \right)^{2} } \right)}},$$

where EAF represents the effect allele frequency (EAF), beta represents the estimated genetic effect on gut microbiome, N represents the sample size, and SE (beta) represents the standard error of the genetic effect. SNPs with F-statistics < 10 were excluded to avoid weak instruments bias^57^.

### Statistical analysis

Five MR analytical methods were implemented to identify the causal associations between gut microbiota and MG using the TwoSampleMR R package in this study^58^, including the IVW, weighted median, MR-Egger, weighted mode and simple mode method. Given that the random-effect IVW method assumes all the genetic variants as valid and combines the Wald ratio for each SNP on the outcome to obtain a pooled causal estimate, it is considered as the most powerful method for MR estimation^57^. Thus, our study applies the IVW as the main statistical analysis. The results of causal associations were presented as odds ratios (OR) and 95% confidence intervals (95% CI). Statistical significance was set at a two-sided *P* value < 0.05. To adjust for multiple testing, false discovery rate (FDR) correction based on the Benjamini–Hochberg method was conducted within each level of the taxonomy tree for the number of taxa tested. At the phylum-level test, results with *p* < 0.05 were considered significant. When assessing the causal relationship between subcategories of each level and MG risk, results with the *P*_FDR_ < 0.1 were considered significant; whereas results with *p* < 0.05 but *P*_FDR_ > 0.1 were considered as nominally significant.

To evaluate any bias of the MR assumptions and ensure the robustness of the above findings, a series of sensitivity analyses were conducted. The Cochrane’s Q test was performed to evaluate the existence of heterogeneity among different instruments, where *p* < 0.05 indicated existing heterogeneity^59^. The asymmetry of the funnel plot indicates the probable directional pleiotropy. The p-value of the intercept in MR-Egger regression was used to assess the horizontal pleiotropic effect, where *p* < 0.05 indicated the presence of horizontal pleiotropy^60^. MR-Pleiotropy RESidual Sum and Outlier (MR-PRESSO) test was conducted to correct horizontal pleiotropy by removing possible outliers^61^. Leave-one-out analysis was performed to detect the influence of a single SNP on the pooled IVW estimates.

### Ethics approval

All the summary data used in this study were obtained from publicly available data sources. Ethical approval and informed consent from the participants were obtained by each cohort enrolled in the original GWAS studies.

### Supplementary Information

Supplementary Tables.

Supplementary Figures.

## Data Availability

The GWAS summary data used in this study are publicly available and further analyses are available from the corresponding author upon request.
