# Supplementary material for: Mendelian randomization study revealed a gut microbiota-neuromuscular junction axis in myasthenia gravis
Source: Sci Rep. 2024 Jan 30;14:2473. doi: 10.1038/s41598-024-52469-7 (PMC10827739; doi:10.1038/s41598-024-52469-7)
Supplement: Supplementary file 2 — Supplementary Figures. [file 41598_2024_52469_MOESM2_ESM.pdf]

Supplementary Figures

Supplementary Fig. 1 Scatterplots of potential effects of SNPs on gut microbiota vs. MG using IVW method, Weighted median method, and MR Egger method.

Supplementary Fig. 2 Funnel plots indicate the probable directional pleiotropy.

Supplementary Fig. 3 Leave-one-out sensitivity analysis of the causal effect of gut microbiota on MG. No single SNP was strongly influencing the overall causal association effects.

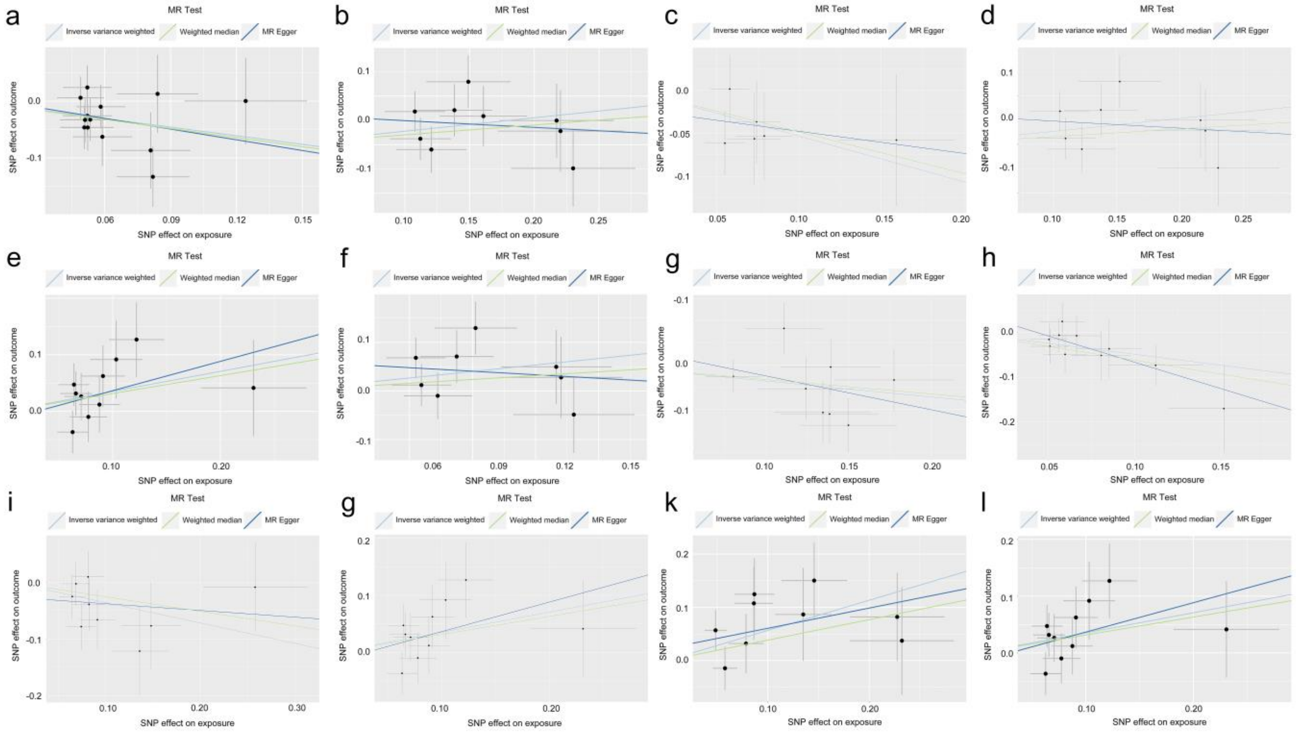

Supplementary Figure 1. Scatterplots of potential effects of SNPs on gut microbiota vs. MG using IVW method, Weighted median method, and MR Egger method. The slope of each line corresponds to the estimated MR effect of each method. (a) phylum Actinobacteria; (b) phylum Lentisphaerae; (c) class Gammaproteobacteria; (d) class Lentisphaeria; (e) order Mollicutes RF9; (f) order Victivallales; (g) family Defluviitaleaceae; (h) family Family XIII; (i) family Peptococcaceae; (j) family unknown family; (k) genus Faecalibacterium; (l) genus unknown genus.

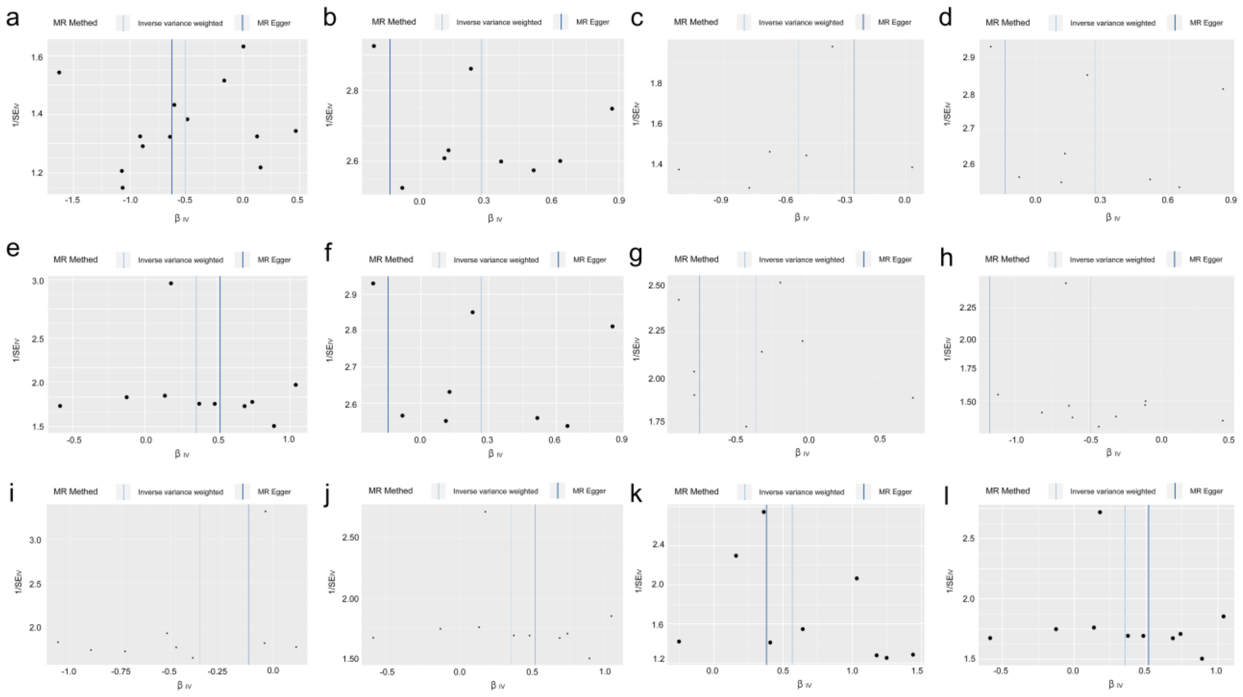

Supplementary Figure 2. Funnel plots indicate the probable directional pleiotropy. (a) phylum Actinobacteria; (b) phylum Lentisphaerae; (c) class Gammaproteobacteria; (d) class Lentisphaeria; (e) order Mollicutes RF9; (f) order Victivallales; (g) family Defluviitaleaceae; (h) family Family XIII; (i) family Peptococcaceae; (j) family unknown family; (k) genus Faecalibacterium; (l) genus unknown genus.

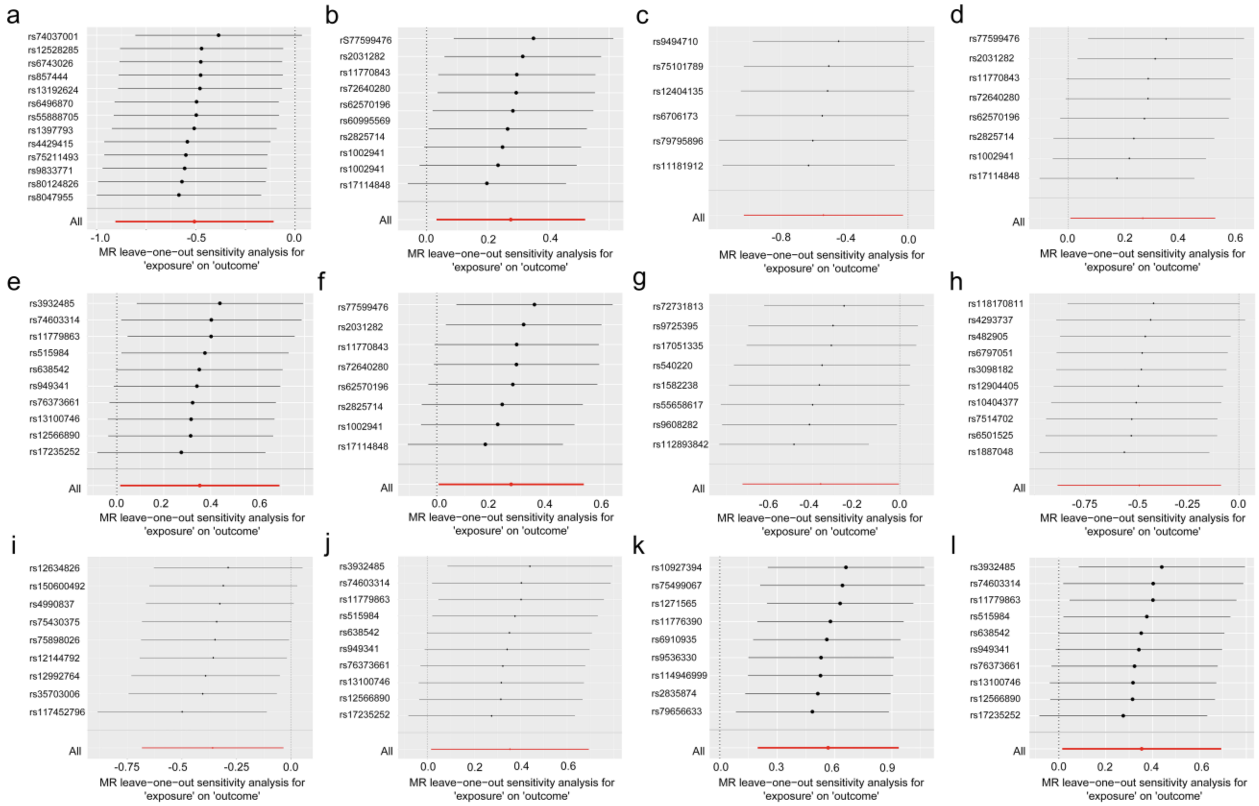

Supplementary Figure 3. Leave-one-out sensitivity analysis of the causal effect of gut microbiota on MG. No single SNP was strongly influencing the overall causal association effects. (a) phylum Actinobacteria; (b) phylum Lentisphaerae; (c) class Gammaproteobacteria; (d) class Lentisphaeria; (e) order Mollicutes RF9; (f) order Victivallales; (g) family Defluviitaleaceae; (h) family Family XIII; (i) family Peptococcaceae; (j) family unknown family; (k) genus Faecalibacterium; (l) genus unknown genus.
